# Craniodentofacial Manifestations in a Rare Syndrome: Orofaciodigital Type IV (Mohr-Majewski Syndrome)

**DOI:** 10.1155/2014/605892

**Published:** 2014-12-21

**Authors:** Meltem Ozdemir-Karatas, Didem Ozdemir-Ozenen, P. Suzanne Hart, Thomas C. Hart

**Affiliations:** ^1^Department of Maxillofacial Prosthodontics, Faculty of Dentistry, Istanbul University, Capa, Istanbul, Turkey; ^2^Department of Pedodontics, Faculty of Dentistry, Yeditepe University, Goztepe, Istanbul, Turkey; ^3^Office of the Clinical Director, National Human Genome Research Institute, National Institutes of Health, Bethesda, MD 20892, USA; ^4^Department of Periodontics, University of Illinois at Chicago, Chicago, IL 60612, USA

## Abstract

*Background.* The orofaciodigital syndromes (OFDS) are a heterogeneous group of syndromes that affect the face, oral cavity, and the digits. OFDS type IV (OMIM %258860) is rare and characterized by broad nasal root and tip, orbital hypertelorism or telecanthus, micrognathia, hypoplastic mandible, and low-set ears. Oral symptoms may include cleft lip, cleft or highly arched palate, bifid uvula, cleft or hypoplastic maxillary and mandibular alveolar ridge, oral frenula, lingual hamartoma, and absent or hypoplastic epiglottis. Dental anomalies are common and generally include disturbances in the number of teeth. *Case Report.* This report presents a six-year-old girl, referred with the chief complaint of missing teeth. She was diagnosed as having OFDS type IV based on clinical findings. Her parents reported three deceased children and two fetuses that had the same phenotype. She was the seventh child of consanguineous parents who were first cousins. *Conclusion.* This is a very rare syndrome. Many reported OFDS type IV cases have consanguineous parents, consistent with an autosomal recessive trait. Manifestation of cleft palate in the healthy sibling may be mild expression of the disorder or an unrelated isolated cleft.

## 1. Introduction

The oral-facial-digital (orofaciodigital) syndromes (OFDS) are a heterozygous group of conditions that affect the face, oral cavity, and the digits. Eleven different types of orofaciodigital syndromes have been reported (OMIM and OFD1–11) [1]. In common to all are facial anomalies, oral findings including cleft or lobulated tongue, oral frenula and/or cleft palate, and digital anomalies including brachydactyly, syndactyly, clinodactyly, and polydactyly. While the presence of other anomalies often makes it difficult to ascertain whether cases with unusual anomalies demonstrate variable expressivity or represent other conditions, they reflect the striking heterogeneity for OFDS. Phenotypic overlap exists between the OFDS. OFDS type IV is also known as Baraitser-Burn syndrome (OMIM %258860) and as Mohr and Majewski syndrome [2]. Clinical findings of both Mohr syndrome (OFDS type II) (OMIM %252100) which is characterized with poly-, syn-, and brachydactyly, lobulated tongue with papilliform protuberances, an angular form of the mandibular alveolar process, supernumerary skull sutures, and an episodic neuromuscular disturbance, and the more severe Majewski syndrome, a lethal recessive disorder characterized by short rib-polydactylyand neonatal chondrodystrophy are described in OFDS type IV [3]. Facial anomalies in OFDS type IV have included broad nasal root and tip, hypertelorism or telecanthus, micrognathia, hypoplastic mandible, and low-set ears. Numerous oral anomalies of this syndrome have been reported including cleft lip, cleft or highly arched palate, bifid uvula, cleft or hypoplastic maxillary and/or mandibular alveolar ridge, oral frenula, lingual hamartoma, and absent or hypoplastic epiglottis. Dental anomalies are common and generally include disturbances in the number of teeth [4–9].

This report presents the craniodentofacial manifestations of a case of OFDS type IV with five deceased siblings with the same phenotype and one sibling with cleft palate only [10].

## 2. Case Report

**Figure 1:**
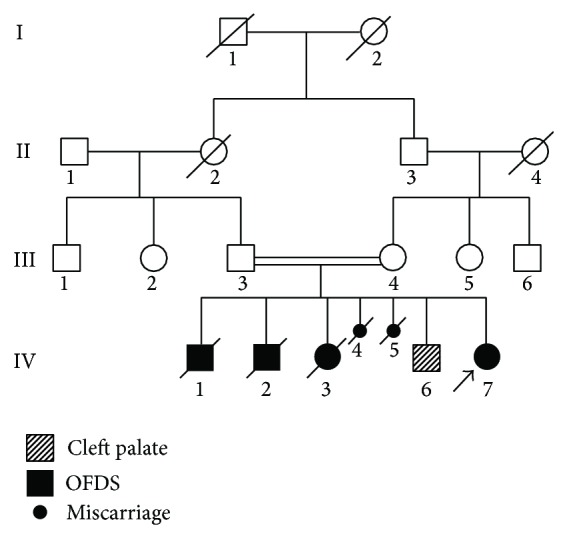
Pedigree of the family (IV-7: proband-with arrow, IV-1, 2, 3: ex siblings with the same phenotype, IV-4, 5: miscarriage, and IV-6: healthy sibling with cleft palate).

A six-year-old girl was referred to Istanbul University, Faculty of Dentistry, Department of Maxillofacial Prosthodontics with a chief complaint of missing teeth. She was diagnosed as having OFDS type IV at the age of 25 days [10]. She was the seventh child of healthy parents who were first cousins (Figure 1). The parents reported that two sons and a daughter died with the same phenotype as the proband but could not provide specifics beyond that each child had oral and digital anomalies. Their two sons were 2- and 5-day old and their daughter was 1-day old at the time of death. The mother had two miscarriages that were reported to have the same phenotype. The other living child of the family (IV-4) had a surgically repaired cleft palate. It is not clear whether this may represent a mild expression of OFDS type IV or unrelated cleft palate.

As the proband's birth certificate indicates, she was born with a birth weight of 2450 gr, length 45 cm, and her head circumference was 32.5 cm (all <3rd centile). At age of 25 days, she was reported to have hypertelorism, depressed nasal bridge, microrethrognathia, median pseudocleft of the upper lip, small and lobulated tongue, pseudocleft of the posterior palate, and bifid epiglottis. She had mesomelic shortness of the limbs with club feet, duplication of great toes, and polysyndactyly. Her radiographs revealed an extra phalanx between the fourth and fifth fingers, bifid thumb, and postaxial digit in the right hand. The left hand radiography showed a forked fifth metacarpal with an extra postaxial digit and her tibias were short and broad. The hemogram report and the urine analysis were normal. The chest radiograph, ECG, abdominal ultrasound, and cranial MR revealed no abnormalities. Hearing loss was detected in her left ear with auditory brain responses. Chromosome analysis using G-banding technique revealed a 46,XX karyotype. At that time, she was diagnosed as having OFDS type IV [10].

The necropsy report of the third child (IV-3) who had died at 1 day of age reported that she had a lobulated, tethered tongue and bifid epiglottis. Her right hand revealed six fingers and thumb was wide and flat. Syndactyly was noted between fourth and fifth fingers. Eight fingers were present on the left hand and the thumb was broad; syndactyly was seen between fourth and fifth, fifth and sixth, and seventh and eighth fingers. Both hands were slightly flexed forward. Mesomelic shortness of the limbs was noticed. She had megaloureter and lobar pneumonia which was reported as the probable cause of death.

## 3. Current Clinical Symptoms

**Figure 2:**
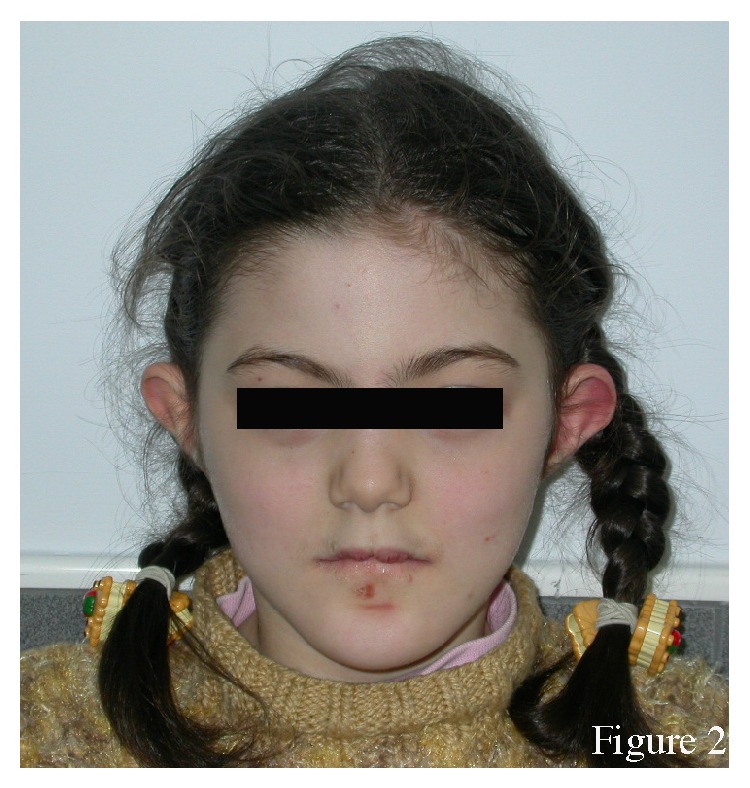
Frontal view of the proband. Note the broad nasal bridge, root, and tip, protruding ears, and bifid frenula.

**Figure 3:**
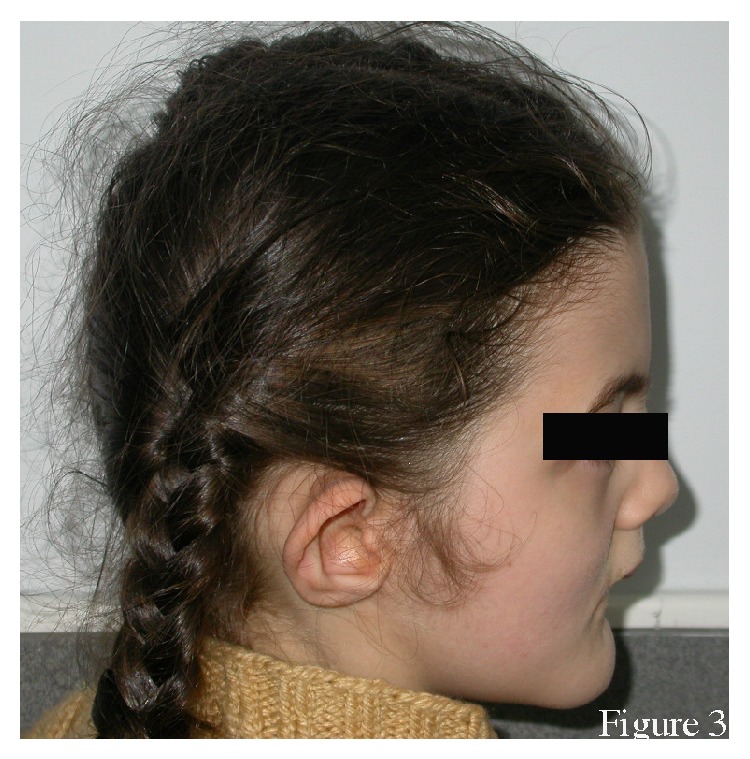
Lateral view of the patient. Note the retrognathic mandible.

**Figure 4:**
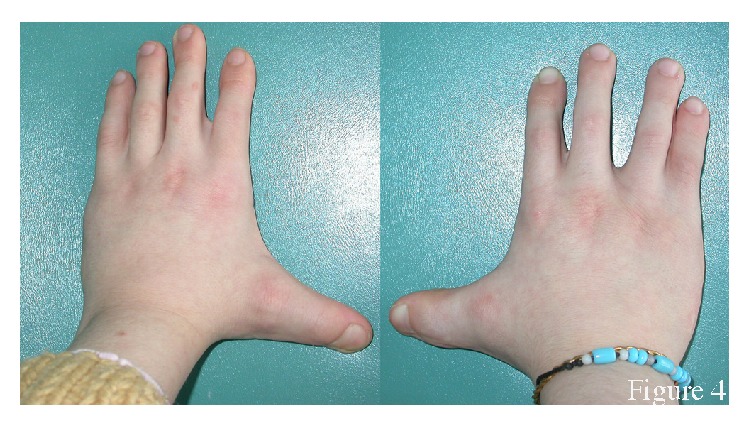
Hands of the patient. Note the surgery scars.

**Figure 5:**
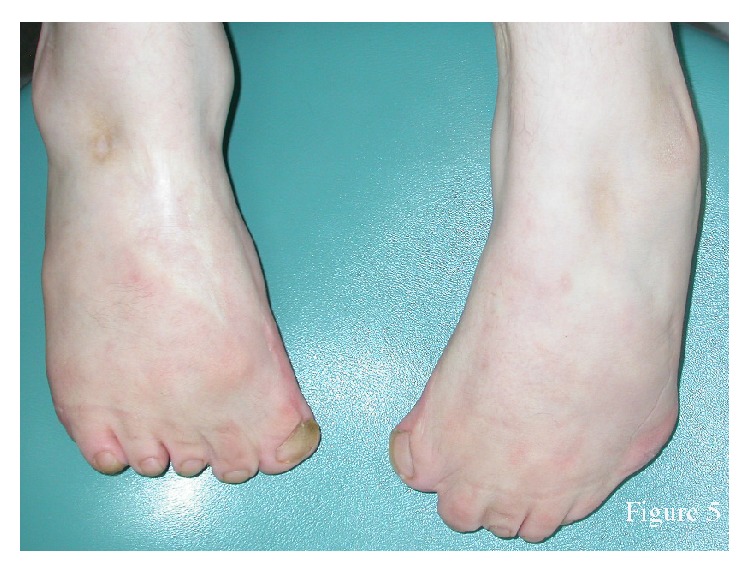
Feet of the patient. Note bilateral talipes equinovarus.

The proband had difficulty walking. She had a broad nasal bridge, root, and tip, protruding ears, and bifid frenula (Figure 2). She had a retrognathic mandible and a severe Angle Class III malocclusion (Figure 3). Cutaneous lesions below her lower lip were suggestive of self-injurious behaviour. She had mental retardation and difficulty talking. She had conductive hearing loss as reported in her medical file. She had broad hands, with syndactyly and polydactyly of both hands, and bilateral bifid thumbs (Figure 4). She underwent multiple surgeries to fix the polydactyly and bifid thumbs. She had bilateral talipes equinovarus (Figure 5). She had undergone many surgeries of hands and feet because of syndactyly, brachydactyly, and polydactyly.

**Figure 6:**
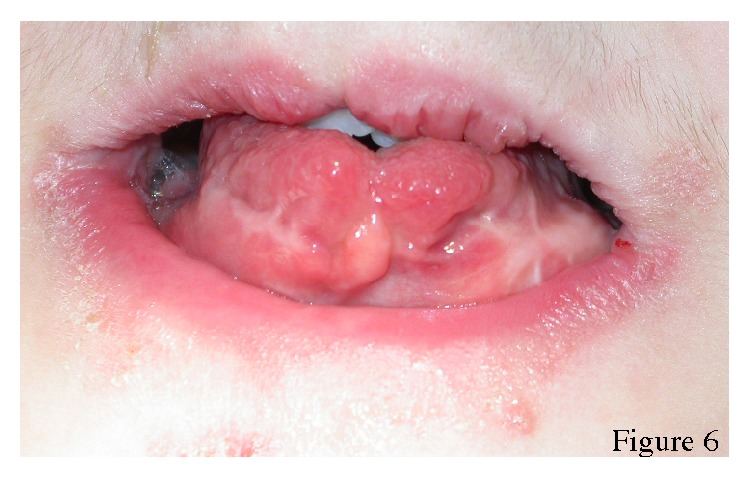
Tongue of the patient. Note the surgery scars secondary to tumor-like lesions.

**Figure 7:**
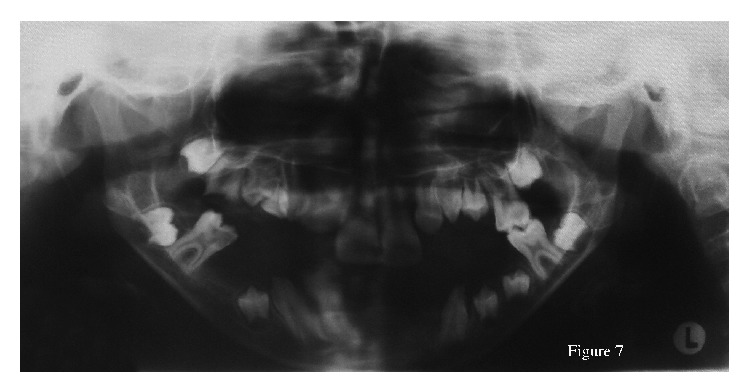
Panoramic radiograph of the patient.

Intraoral examination revealed cleft palate and a pseudo-cleft lip, sublingual hamartoma, and lobulated tongue that had been operated on bilaterally because of tumor-like lesions (Figure 6). Dental findings included the presence of profound carious lesions on the permanent upper and lower right first molars and superficial dentine carious lesions on the permanent upper centrals and both left first molars. All the primary teeth were exfoliated, all the permanent first molars, upper centrals, and left upper and right lower lateral teeth were erupted. Her panoramic radiograph revealed oligodontia with congenitally absent upper permanent second premolars, the lower permanent central incisors, the left lateral incisor, and the right second premolar (Figure 7). Dental treatments, performed with local anaesthesia, consisted of placement amalgam restorations for the left upper and lower permanent first molars and root canal treatment for the carious right first molar. The root of right upper permanent first molar was extracted.

## 4. Discussion

The OFDS are rare and demonstrate considerable clinical heterogeneity, making definitive diagnosis problematic. This case presented with findings of both OFDS types II and IV. The lobulated tongue with hypertrophied frenula, median pseudocleft of the upper lip, and hearing loss are findings characteristic of OFDS type II whereas the tibial dysplasia and club foot deformities are characteristic of OFDS type IV. Both conditions are reported to show overlapping oral findings including high arch palate, cleft palate, and lobulated tongue with nodules. Temtamy and McKusick [11] first reported the overlapping features of the Mohr and Majewski Syndromes in two cases (Table 1). They proposed that Mohr and Majewski syndromes may be mild and severe expressions of the same disorder. Baraitser et al. [12] reported a female infant with features of Mohr syndrome (OFDS type II) and Majewski syndrome (short rip polydactyly syndrome type II) who was the second child of first cousin parents. Clinical features of their patient are shown in Table 1.

In their review of OFD syndromes, Toriello classified the syndrome into 11 groups as types I through IX, Mohr-Majewski syndrome, and Egger-Joubert syndrome [13]. Mohr syndrome was first defined by Mohr in 1941 with a male patient who had high-arched palate, a lobulated tongue with papilliform outgrowths, a broad nasal root and ocular hypertelorism, syndactyly, brachydactyly, and polydactyly [14]. In 1984, Burn et al. reported an affected second cousin of the patient of Baraitser with suggested autosomal recessive inheritance [15]. Their patients were presented at the European Society of Paediatric Radiology in Paris in 1983 and the proposed designation OFDS type IV was accepted. Subsequent cases have been reported and clinical summaries are provided in Table 1. Most of these cases were diagnosed at pregnancy and the pregnancies were terminated. Parental consanguinity is reported in most of the OFDS type IV cases reported in the literature. This raises the possibility that part of the phenotypic spectrum reported for OFDS type IV may arise from the contribution of homozygous genetic loci at different chromosomal locations, independent of the gene(s) that contribute to the underlying OFDS condition.

Most of the reported clinical findings for OFDS type IV cases come from necropsy reports of fetuses. The proband reported here is six-year old and has all characteristic features of the syndrome. Dental findings of oligodontia and significant caries were also noted. Conductive hearing loss which is also a characteristic of OFDS type IV was present in this case. As the parents are consanguineous, transmission of the syndrome in this family is also consistent with an autosomal recessive trait. The presence of six and possibly seven affected offspring in the same family provides a unique opportunity to see the phenotypic variability of this condition. While family reports support autosomal recessive transmission of the condition, it is unknown if OFDS type IV results from mutation of a single gene locus or if locus heterogeneity contributes to the observed clinical variability.

## 5. Conclusion

There are only sixteen patients reported in the literature with this syndrome, and few report dental findings and report longitudinal followup describing developmental progression of OFDS type IV. The palatal pseudocleft reported in infancy has progressed to a frank palatal cleft. Most OFDS type IV cases report consanguineous parents. In the current family, both affected males and females were observed, consistent with autosomal recessive inheritance of the trait. The affected individuals also demonstrate the striking heterogeneity reported for the condition, ranging from neonatal lethality to infant lethality to a fully affected OFDS type IV sibling. The male sibling with cleft palate may represent a mildly affected spectrum of the OFDs Type IV phenotype.

## 6. Why This Paper Is Important

Of the eleven OFDS types reported, OFDS type IV cases are very rare, and the range of clinical heterogeneity is unclear. This case reports longitudinal followup of an OFDS case first reported in early infancy. The palatal pseudocleft has progressed to a frank palatal cleft, and additional dental findings are reported. The proband had one healthy sibling with a cleft palate. It is thus concluded that the inheritance of this syndrome is most consistent with an autosomal recessive trait and the cleft palate of the healthy sibling may reflect a mild expression of the syndrome. Although this syndrome is referred to as Baraitser-Burn syndrome in the OMIM database, consideration of all clinical reports is most parsimonious with the OFDS type IV Mohr-Majewski syndrome.

## Figures and Tables

**Table 1:** Clinical features of current family and patients reported in the literature.

Anomaly	IV-3	IV-7	1 [11]	2 [11]	3 [12]	4 [15]	5 [16]	6 [16]	7 [17]	8 [18]	9 [2]	10 [19]	11 [20]	12 [21]	13 [22]	14 [22]
Orofacial																
Frenula	−	+	+	+	+	+	+	+	−	−	+	−	−	?	+	+
Lobulated tongue	+	+	+	?	−	−	+	+	+	+	+	+	+	+	+	+
(Pseudo) cleft lip	−	−	+	+	−	−	−	−	+	+	−	−	−	+	+	+
Cleft palate	−	+	−	−	+	−	−	+	+	+	+	+	+	+	+	+
Micrognathia	−	+	+	+	+	+	+	+	+	+	+	+	+	?	+	+
Hypertelorism	−	+	+	+	+	+	+	+	+	+	+	−	?	?	+	+
Broad nasal tip	−	+	?	+	+	+	+	+	+	+	+	?	?	?	?	?
Dental anomalies	?	?	?	?	?	?	+	?	+	?	?	?	?	?	?	?
Hand																
Preaxial polydactyly	+	−	−	−	−	−	−	+	+	+	−	−	−	−	+	+
Postaxial polydactyly	+	+	−	−	+	+	+	+	+	+	+	+	+	+	+	+
Syndactyly	+	+	−	−	+	+	+	+	−	+	+	+	+	+	+	+
Foot																
Preaxial polydactyly	+	+	+	+	+	+	+	+	+	+	+	+	−	−	+	+
Postaxial polydactyly	+	+	+	−	+	+	+	+	+	+	+	+	−	−	+	+
Clubfoot	+	−	−	−	+	+	+	+	+	+	−	+	−	−	−	−
Skeletal																
Short ribs	−	−	+	−	−	−	−	−	−	−	−	+	−	+	−	−
Short tibia	+	+	+	+	+	+	−	−	+	+	+	+	+	+	+	+
Short radius/ulna	−	−	?	?	−	−	−	−	−	−	+	+	+	+	+	+
Visceral																
Heart defects	−	+	+	?	?	?	−	−	+	−	+	−	−	+	+	+
Renal defects	+	+	?	?	?	?	−	?	−	−	+	+	+	+	−	−
Larynx hypoplasia	−	+	?	?	+	+	+	+	−	−	+	+	−	−	+	+
Epiglottis hypoplasia	−	+	+	?	+	+	+	+	+	−	+	+	+	−	+	+
Larynx hamartoma	−	−	?	?	?	?	+	?	−	−	−	−	−	−	+	+
CNS																
Brain malformation	−	+	?	?	+	?	?	?	?	?	+	+	+	+	+	+
Mental retardation	?	+	?	?	+	+	+	?	?	?	?	?	?	?	?	?
Other																
Ambiguous or hypoplastic genitalia	−	−	?	+	−	−	−	−	−	−	+	+	−	−	+	+
Deafness	?	+	?	+	+	+	?	?	+	?	?	?	+	?	?	?
Early death	Yes	No	Yes	No	No	No	No	?	Yes	Yes	Yes	Yes	Yes	Yes	Yes	Yes
